# Characterization of scotopic and mesopic rod signaling pathways in dogs using the On–Off electroretinogram

**DOI:** 10.1186/s12917-022-03505-z

**Published:** 2022-12-03

**Authors:** Nate Pasmanter, Simon M. Petersen-Jones

**Affiliations:** Department of Small Animal Clinical Sciences, College of Veterinary Medicine, Michigan State University, 736 Wilson Road, D208 East Lansing, MI USA

**Keywords:** ERG, Long flash, On–off, Dogs, Rods

## Abstract

**Background:**

The On–Off, or long flash, full field electroretinogram (ERG) separates retinal responses to flash onset and offset. Depending on degree of dark-adaptation and stimulus strength the On and Off ERG can be shaped by rod and cone photoreceptors and postreceptoral cells, including ON and OFF bipolar cells. Interspecies differences have been shown, with predominantly positive Off-response in humans and other primates and a negative Off-response in rodents and dogs. However, the rod signaling pathways that contribute to these differential responses have not been characterized. In this study, we designed a long flash protocol in the dog that varied in background luminance and stimulus strength allowing for some rod components to be present to better characterize how rod pathways vary from scotopic to mesopic conditions.

**Results:**

With low background light the rod a-wave remains while the b-wave is significantly reduced resulting in a predominantly negative waveform in mesopic conditions. Through modeling and subtraction of the rod-driven response, we show that rod bipolar cells saturate with dimmer backgrounds than rod photoreceptors, resulting in rod hyperpolarization contributing to a large underlying negativity with mesopic backgrounds.

**Conclusions:**

Reduction in rod bipolar cell responses in mesopic conditions prior to suppression of rod photoreceptor responses may reflect the changes in signaling pathway of rod-driven responses needed to extend the range of lighting conditions over which the retina functions.

## Background

The mammalian retina is uniquely equipped to process visual signals across a substantial range of luminances. In the dark, photoreceptors in the outer retina maintain a relatively depolarized state, with passive and active transport of cations in the outer segments causing an electrical current to flow along the length of the photoreceptor. In rods, this is known as the dark current [1–3]. In the dark-adapted retina, both rods and cones can respond to light stimulus; with weak light stimuli, the response is rod-driven – with stronger flashes, there is a mixed rod-cone response. Progressive increases in background light desensitize and suppress the rod response, such that the light-adapted retinal response is cone-driven [4–7].

The visual signal is shaped by complex retinal processing that divides into two parallel pathways – ON and OFF. The separation of these pathways begins with ON and OFF bipolar cells, second order neurons in the retina that synapse with rod and cone photoreceptors [8–10]. Bipolar cells are classified based on their response to light stimulus of the photoreceptors – ON bipolar cells, including rod bipolar cells (RBCs), depolarize, whereas OFF bipolar cells hyperpolarize, in response to a light stimulus driven decrease in glutamate release from photoreceptor synaptic terminals [11, 12]. The bipolar cell response is further shaped by photoreceptor pathways; cones synapse with both ON and OFF cone bipolar cells, whereas rods primarily interact with RBCs when responding to weak stimuli, but have additionally been shown to signal via gap junctions with cone photoreceptors as well as by direct connections with OFF cone bipolar cells [13–17]. The alternative rod pathways are more prominent in mesopic conditions as well as in response to higher frequency flickering light stimuli [18–20]. Horizontal cells are also involved in processing the visual signal but because of their orientation in the retina do not make a significant contribution to the electroretinogram as recorded on the corneal surface.

The separation of flash On- and Off-responses with the full-field ERG provides a useful tool for the characterization of postreceptoral responses. The flash On- and Off ‘-responses’ are separate from ON and OFF pathways. The On-response is the retinal response to flash onset, beginning with photoreceptor hyperpolarization (generating the major portion of the a-wave) and leading to the depolarization of ON bipolar cells (which is the driver of the positive b-wave) as well as hyperpolarization of OFF bipolar cells (which has contributions to both the shape and amplitude of the a- and b-waves, particularly the early portion of the light-adapted a-wave in primates) [3, 15, 21–24]. In contrast, the Off-response is the retinal response to stimulus offset, and is generated by several components – in humans, an initial rapid positive deflection (the d-wave) is generated primarily by OFF bipolar cells, but there are additional contributions from photoreceptors (which return to a relatively depolarized state resulting in a slow cornea-positive response) and ON bipolar cells (hyperpolarize, resulting in a fast cornea-negative response) [25–29]. With short-duration flashes these responses are merged in the ERG, and the recorded waveform reflects the combined contribution of these components. Additionally, short-duration flash cone responses exhibit a ‘photopic hill’ effect whereby with increasing stimulus strength the cone-driven b-wave reach a maximal amplitude and then decreases with widening of the b-wave and lengthening of the peak time. This occurs as the Off pathway response slows and separates from that of the On pathway meaning the two responses are temporally separated rather than being superimposed [13–15].

The component waveforms of the ERG are shaped by ‘processes’ with contributions from different retinal cells (named PI/PII/PIII by Granit based on the order of disappearance under anesthesia) [30]. A major focus of this paper are the changes in response of PIII, which is driven by photoreceptors and is the primary contributor to the cornea-negative a-wave, and PII, driven mainly by ON bipolar cells (but additionally shaped by OFF bipolar cells) that heavily influences the cornea-positive b-wave. Note that PIII is present for the duration of a sustained flash and returns to baseline at flash offset, whereas PII differs at flash onset or offset based on the relative contributions of the ON and OFF pathways [11, 12, 31]. These processes have been shown to further differ in humans and rats based on background luminance, with a greater decline in the amplitude of PII relative to the rod-driven PIII with increasing background luminance [23, 32].

There are interspecies differences in the ERG Off-response (at flash offset). Two broadly different types of flash Off-responses have been identified in mammals as first described by Granit and Therman in 1935—the E-type retina in species such as the rat and mouse, and I-type retina of humans and other primates [25, 27, 33]. The I-type retina has a primarily positive d-wave at termination of the stimulus (Off response) coupled with a relatively large photopic a-wave amplitude at stimulus onset. In contrast, the E type retina has a primarily negative response at the termination of the stimulus and a relatively small a-wave as part of the On-response. Isolation of the receptor response (PIII) by administration of aspartate shows that there is the PIII waveform is maintained during stimulation with a gradual return to baseline, in response to sustained flashes, and thus current generated by photoreceptors are unlikely to explain this difference in the Off-response [34–37].

Few studies have addressed canine responses to the On–Off ERG, although the technique has been used in the study of some canine inherited retinal degenerations. The dog has emerging importance as a model for development of translational therapy for human conditions. Inherited retinal disease models are more common in dogs than cats—therefore there is a need to fully understand the components of the dog ERG with conditions such as CSNB being recognized in the dog [38–44]. The dog exhibits a predominantly negative Off-response of the E-type retina [45]. The purpose of this study was to determine baseline features of the On- and Off-response in phenotypically normal dogs as well as to assess postreceptoral pathways and changes with increasing background luminance in the canine retina.

## Results

### Characterization of the canine On–Off ERG

**Fig. 1:**
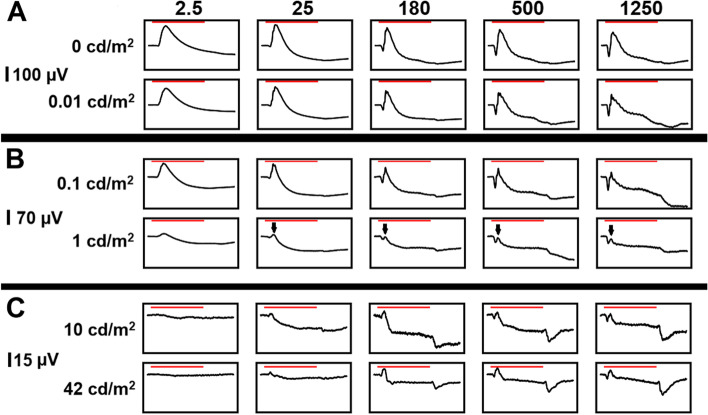
Representative On–Off series ERG tracings with a range of background luminances. In each instance responses to 2.5, 25, 180, 500 and 1,250 cd/m2 stimuli presented for 250 ms are shown (red line above tracing indicates duration of stimulus). **A** responses with 0 (no background) and 0.01 cd/m^2^ background. **B** responses with 0.1 and 1 cd/m^2^ background. The small b-wave superimposed on a longer negative deflection is denoted by arrows in the 1 cd/m^2^ background. **C** responses to 10 and 42 cd/m^2^ background. Note the amplitude scale difference between the three panels

Our protocol was designed to examine rod-only, cone-only, and mixed rod-cone contributions to the On–Off ERG using increasing background luminance. Representative tracings of a series of 5 stimulus strengths (250 ms stimuli of 2.5, 25, 180, 500 and 1250 cd/m^2^) superimposed on 4 background white light luminances (no light, 0.01, 0.1, 1, 10 and 42 cd/m^2^) are shown in Fig. 1. In the presence of none or low background light levels the ERG response was predominantly rod-driven – with increasing background luminance the rod contribution was sequentially decreased. We consider the response on a background luminance of 42 cd/m^2^ to be a cone only response (typically 30 cd/m^2^ is considered to be a rod-suppressing background). Although preceded by a small positive deflection, the Off-response in the dog was predominantly negative in all stimulus and background conditions.

The shape and amplitudes of the waveforms differed considerably with both stimulus strength and background luminance. Under none or low background light the b-wave was prominent as was the a-wave in response to stronger stimuli. In response to weaker stimuli the downslope following b-wave peak was prolonged, but this became faster with increasing stimuli. With dimmer background light levels there was minimal change in the waveform at stimulus cessation (the Off-response). With increasing background light levels the b-wave amplitude was reduced, for example, in the presence of a 1 cd/m^2^ background light the waveform had an initial negative component with a small positive b-wave component (indicated by arrows in Fig. 1B) superimposed on the down slope. With stronger stimuli and increasing background luminance an Off-response became more prominent. The Off-response had a small positive component (which was most apparent for the three strongest flash stimuli in the 10 and 42 cd/m^2^ background recordings likely reflecting predominance of the cone-driven contributions) followed by a larger negative component. With increasing stimulus strength there was less of a negative post b-wave component.

**Fig. 2:**
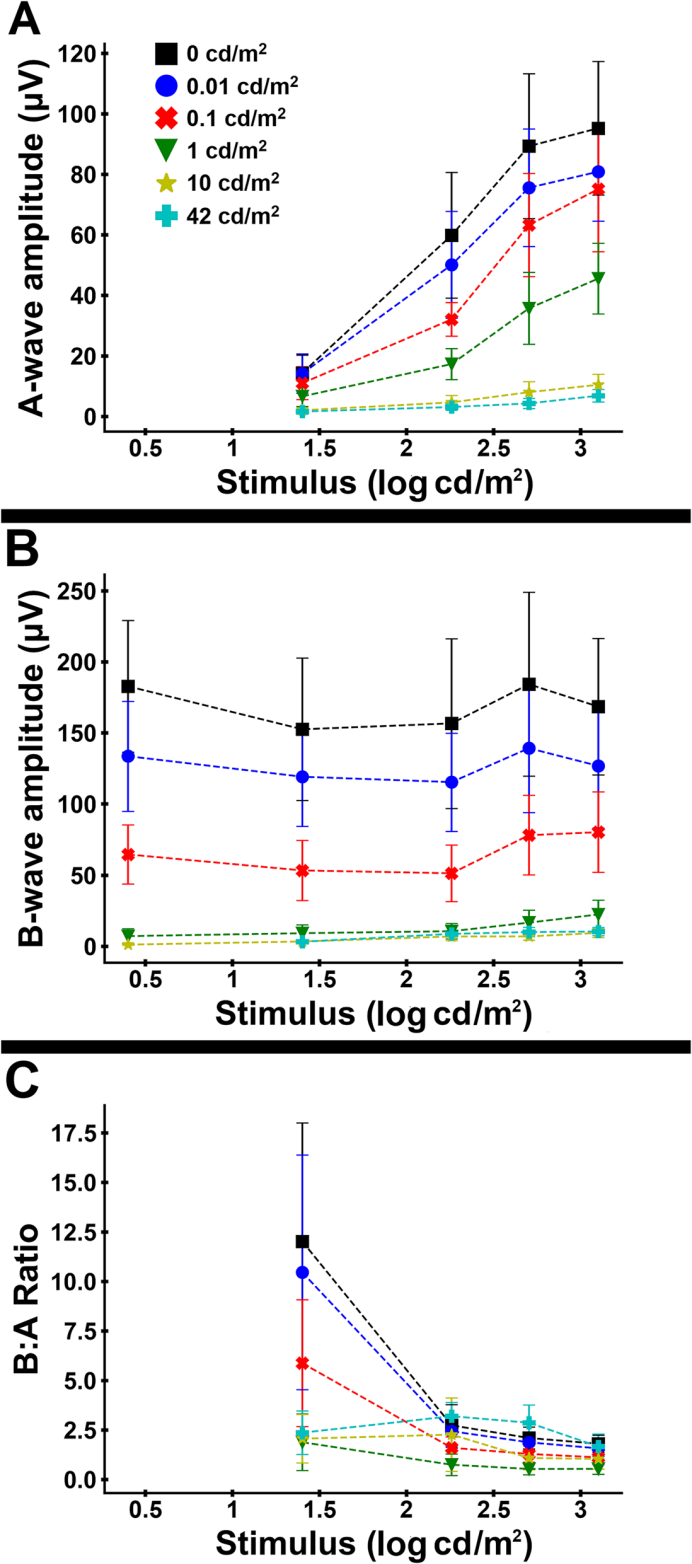
Variation in mean (± standard deviation) On-response amplitudes with stimulus strength and background luminance. **A** Mean a-wave amplitude. **B** Mean b-wave amplitude. **C** Ratio of B:A-wave amplitudes. Different colors and symbols are used to denote the different background luminances

A-wave amplitudes increased with increasing stimulus strength showing semi-saturation kinetics and declined with increasing background luminance (Fig. 2A). In contrast, the b-wave amplitudes were relatively constant with increasing stimulus strength (Fig. 2B). The b-wave amplitudes showed a substantially greater decline with increasing background luminance compared to the a-wave – this led to large decreases in the b:a ratio between 0 and 1 cd/m^2^ background luminance (Fig. 2C). This suggests that the postreceptoral components of the rod On pathway are suppressed at dimmer background luminances than the negative waveform (PIII response which originates directly from rod photoreceptors).

**Fig. 3:**
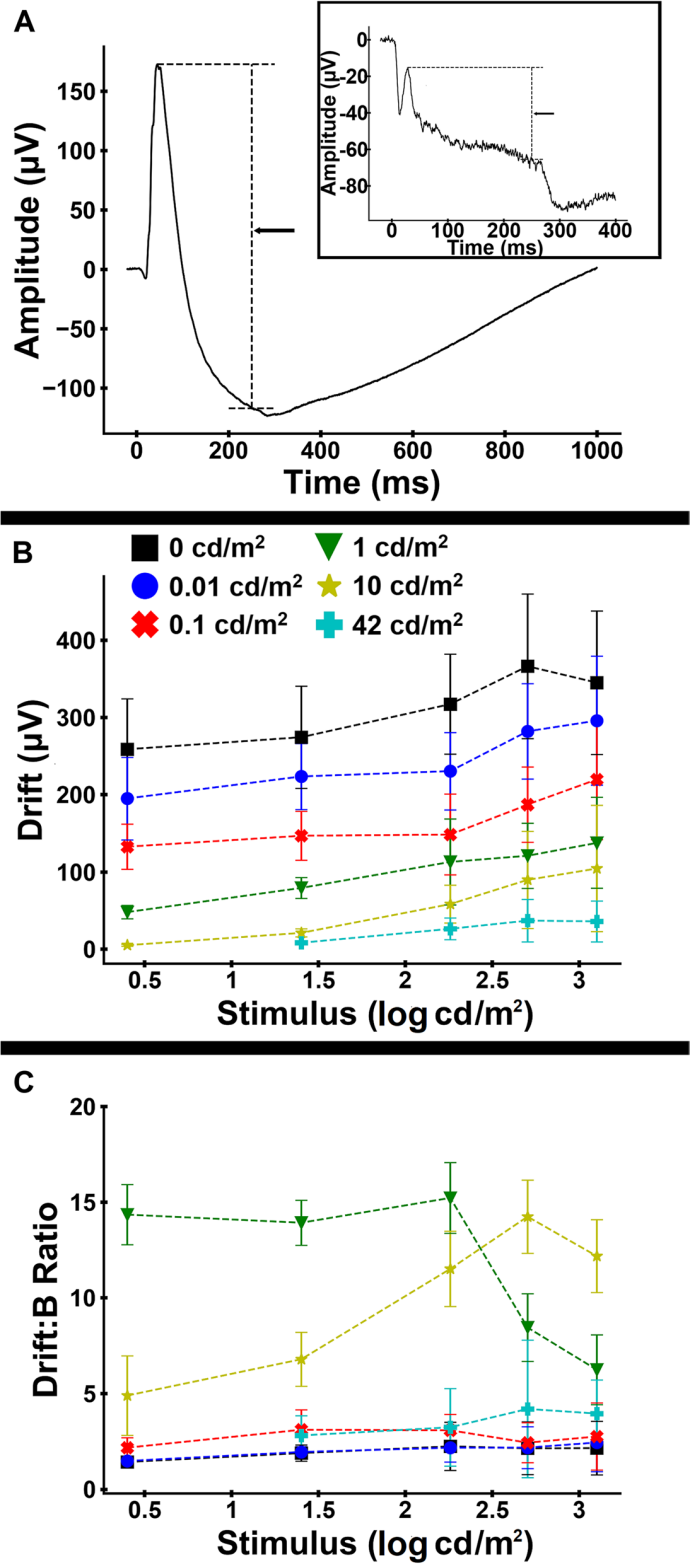
Variation in drift with stimulus strength. **A** Demonstration of the measurement of ‘drift’—change in amplitude between the peak of the b-wave and amplitude at flash cessation, immediately prior to the Off-response component. See the inset for another example with a stronger background luminance condition. **B** Mean (± standard deviation) of drift amplitude. **C** Mean (± standard deviation) drift:b-wave ratio. Different colors and symbols are used to denote the different background luminances

**Fig. 4:**
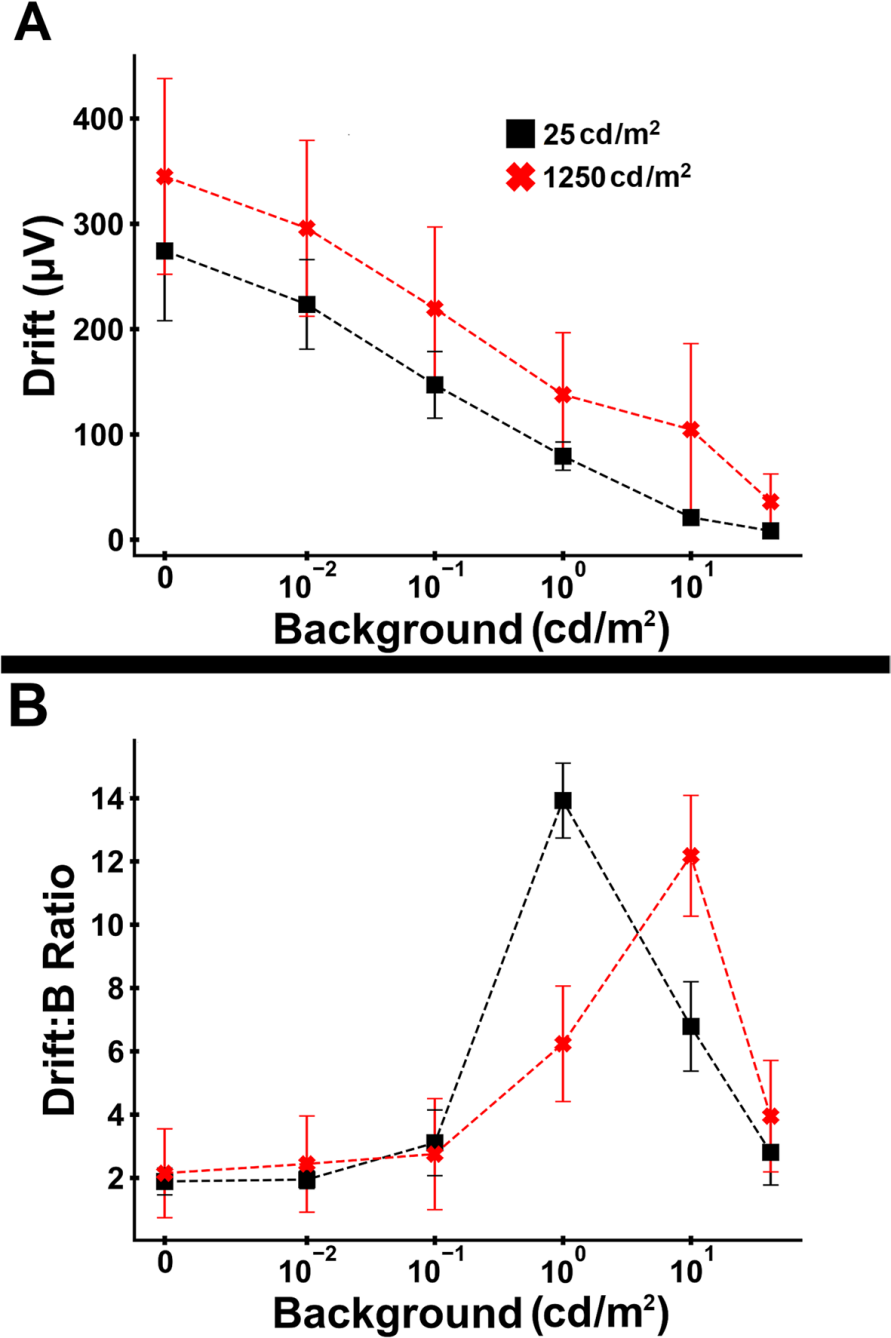
Variation in drift with background luminance for stimulus luminances of 25 and 1,250 cd/m^2^. **A** Mean (± standard deviation) drift amplitude. **B** Mean (± standard deviation) drift:b-wave ratio. Different colors and symbols are used to denote stimulus strength

There was a similar phenomenon with change in the underlying negativity to the waveform. We measured the ‘drift’ amplitude, which we defined as the absolute change between the peak of the b-wave and amplitude at flash cessation prior to the d-wave (see Fig. 3A). The increase in drift amplitudes coupled with a decline in b-wave amplitudes resulted in an increase in the drift:b-wave ratio between 0 and 1 cd/m^2^ background luminance (Figs. 3B-C & 4). There was also a striking difference between the drift:b-wave ratio between two brightest background luminances (Fig. 3C; 10 cd/m^2^ background in yellow and 42 cd/m^2^ background in cyan), which may indicate continued rod contributions to the 10 cd/m^2^ background ERG. Furthermore, the drift:b ratio peaked between 1 and 10 cd/m^2^ background luminance, depending on stimulus strength, and declined with the strongest background luminance (Fig. 4).

**Fig. 5:**
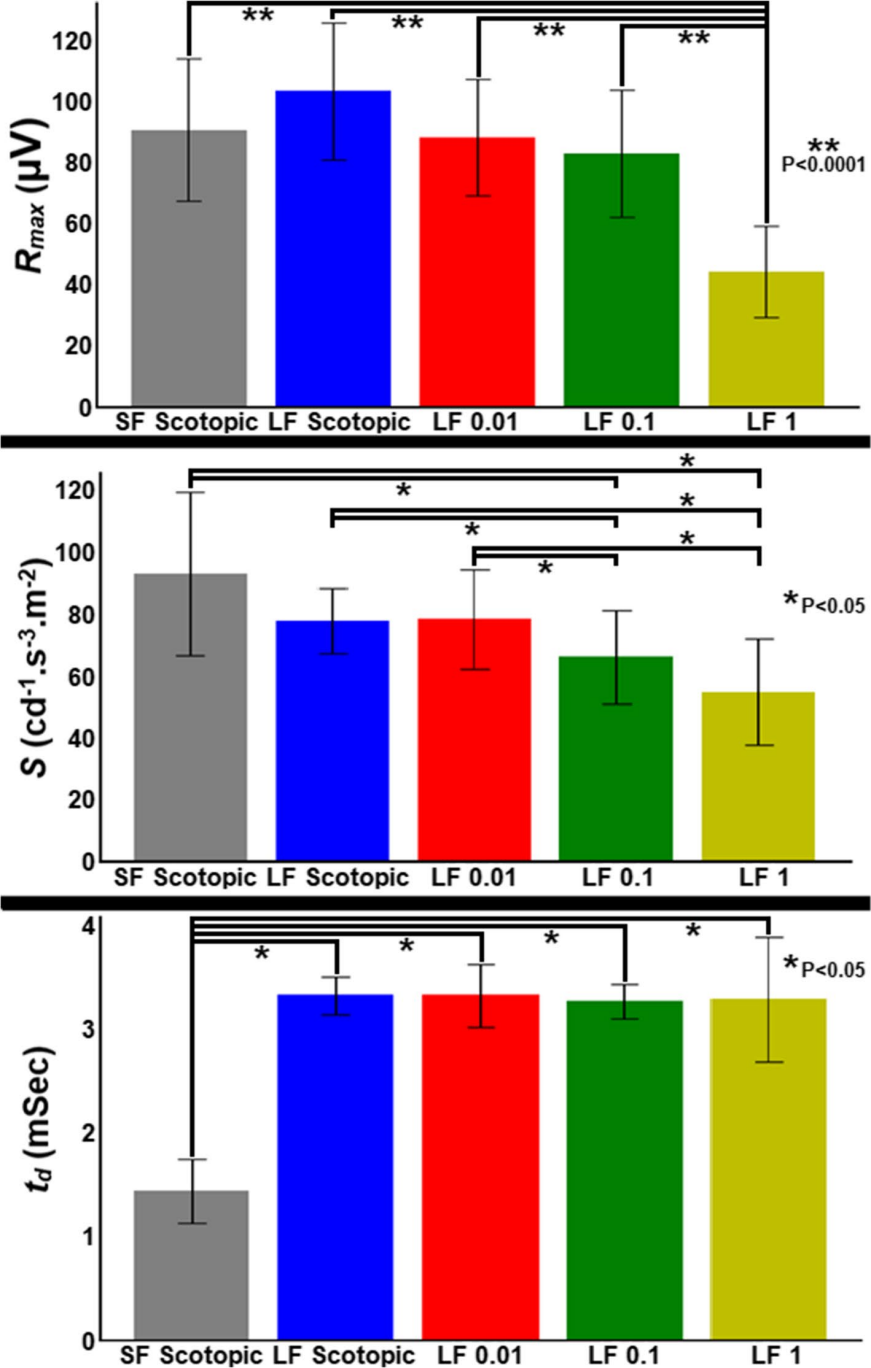
Changes in a-wave model parameters for short flash and On–Off response to no background (dark-adapted) and 0.01, 0.1 and 1 cd/m^2^ background luminance. **A** Mean (± standard deviation) *R*_*max*_*.* **B** Mean (± standard deviation) sensitivity (*S*). **C** Mean (± standard deviation) time delay (*t*_*d*_). ‘SF’ and ‘LF’ are used as abbreviations for short flash and long (On–Off) flash, respectively, and the corresponding background luminance is provided after these. Parameters are derived using the Birch and Hood formula following subtraction of fully light-adapted responses to reveal the rod response

To better characterize photoreceptor contributions to the On-response, we fit an equation described by Birch & Hood to the leading edge of the rod a-wave for background luminances of no background, 0.01, 0.1 and 1 cd/m^2^. For these calculations, the model first subtracted the response at 42 cd/m^2^ to remove cone components (essentially the same photopic subtraction used when modeling the a-wave of the short flash ERG). Model parameters are also included from short-flash ERGs for comparison in Fig. 5. The model demonstrated similar changes in the receptor maximum response, *R*_*max*_ (Fig. 5 – first row) and sensitivity, *S* (Fig. 5 – second row) parameters with increasing background luminance, up to 0.1 cd/m^2^ although there was a reduction in *R*_*max*_ in the 1 cd/m^2^ background. *R*_*max*_ and *S* parameters of the short flash and On–Off scotopic ERGs were similar. However, we did find a substantial difference in the time delay parameter, (*t*_*d*_) which was higher for the On–Off compared to short flash ERG (Fig. 5 – third row). This suggests that there is a part of the flash-offset response that contributes to the a-wave that is not present with the longer flash duration.

**Fig. 6:**
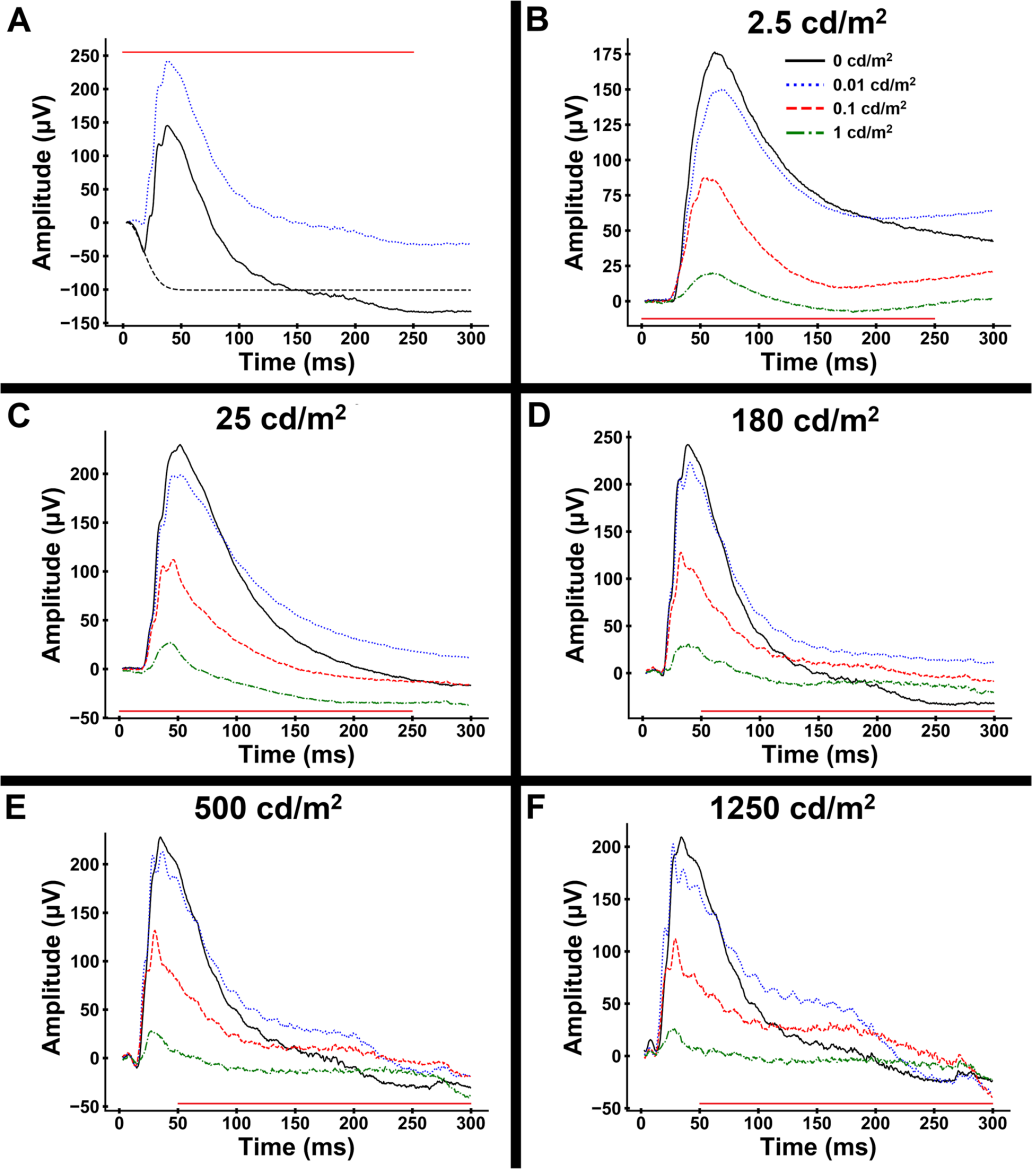
Isolating the PII response. **A** The rod modeled PIII response (black dashed line) is subtracted from the rod response (following subtraction of the cone (photopic) response) (black line) to give the PII response (shown in **A** for the 180 cd/m^2^ flash stimulus on the 0 cd/m^2^ background). The method is applied to the ERG at 0 (no background) 0.01, 0.1, 1 cd/m^2^ backgrounds with stimuli strengths of **B** 2.5 cd/m^2^. **C** 25 cd/m^2^. **D** 180 cd/m^2^. **E** 500 cd/m^2^. **F** 1,250 cd/m^2^. Representative results are shown, and background luminance is denoted by different colors and line styles as indicated in the legend in B

We further analyzed the differences in a-wave model parameters using a repeated-measures ANOVA to compare across the different luminance conditions. Between group comparisons for all three parameters (*R*_*max*_, *S*, and *t*_*d*_) were significantly different (at a *p* < 1 × 10^–4^ level). We then performed a post-hoc Tukey HSD test on each parameter. For the *R*_*max*_ parameter, the amplitude of the 1 cd/m^2^ luminance was significantly lower than the other four luminance conditions (*p* < 0.05). For the *S* parameter, the sensitivity of both the 0.1 and 1 cd/m^2^ luminance were significantly lower than the other three luminance conditions (*p* < 0.05). For the *t*_*d*_ parameter, the time of the short flash condition was significantly faster than the other four luminance conditions (*p* < 0.05). To further interrogate postreceptoral rod pathways, particularly the large negative response seen most obviously in the responses with a 1 cd/m^2^ background, we isolated the PII response by subtracting the modeled PIII process (as shown in Fig. 6A). With increasing background the isolated PII response decreased (Figs. 6B-F). This demonstrated that the negative shape to the waveform with the 1 cd/m^2^ background luminance (which was also present to a lesser extent with the 0.1 cd/m^2^ background) was mainly attributable to the rod-driven PIII component. Furthermore, the negative ‘drift’ (as defined above) present at all backgrounds was essentially eliminated in the isolated PII response, which further supports that the negativity present with sustained flash (as compared to short flash stimuli) is driven by sustained rod activation.

Examination of the isolated PII component revealed further differences with increases in both stimulus strength and background luminance. Although the peak amplitude of both the 0 (no background) and 0.01 cd/m^2^ background recordings were similar across all tested stimuli, there was an evident shift to a shorter peak time with the brighter background. This was also apparent in the 0.1 and 1 cd/m^2^ background recordings in addition to substantial declines in amplitude. We also observed a narrowing (time between the beginning of the leading slope and return to baseline) of the isolated PII response with increasing background luminance, which may reflect a shift in rod signaling pathways.

## Discussion

The light-adapted Off-response in the dog has a small positive component (d-wave) but is predominantly negative. The amplitude of this response scales with stimulus strength and background luminance (see Fig. 1). This shape of Off response is similar to that of the rat and in contrast to that of primates where a more prominent positive d-wave is present. This difference is probably due to differences between the species in the relative contributions of ON and OFF pathways. The cessation of ON bipolar cell driven responses as they hyperpolarize with cessation of the light stimulus to the photoreceptors probably drives the negative Off-response in the On–Off ERG. The relatively small positive d-wave in the dog Off-response suggests that the OFF bipolar cell contributions towards shaping the waveform are relatively small in this species. When extrapolated to the On-response it also seems likely that OFF bipolar cells make less of a contribution to the photopic a-wave of the dog than they do in primates [29]. These findings are further supported by a previously characterized canine model of complete CSNB, wherein the light-adapted On–Off ERG in affected dogs demonstrated a substantially more positive Off-response compared to control dogs (thus indicating a relatively greater role of the ON pathway in the normal canine retina) [46].

The amplitude and shape of the On–Off ERG changes with increases in background luminance. When moving from dark-adapted to partial light adaptation the photoreceptor-driven a-wave has a significantly slower decline in amplitude compared to the postreceptoral b-wave of the On-response. This disparity results in a ‘negative type’ ERG appearance (a negative ERG being one where the b-wave is smaller than the a-wave). Similar findings have been reported in human studies of the short-flash ERG and have been posited to reflect a mechanism for maintenance of retinal sensitivity across a wide range of luminance [47–50]. The findings reported here suggest that there is a similar occurrence in dogs.

We applied the Birch & Hood model of the rod-driven a-wave to parameterize the PIII response in the no background and low luminance backgrounds. The most significant difference in model parameters compared to the regular flash ERG was seen in the ‘time delay’ parameter *t*_*d*_ which was greater in the On–Off ERG. There were smaller decreases in the amplitude parameter *R*_*max*_ that mirrored the changes in a-wave amplitude with increasing background luminance. As the dog ERG has relatively small photopic amplitudes (compared to humans), our results support the conclusion that the responses are primarily rod-driven and a reduction in rod responses leads to a commensurate decrease in a-wave amplitudes. Additionally, the time delay parameter is effectively the time from flash onset to beginning of the a-wave – so while there is likely some component of the Off-response that drives the initial slope of the a-wave, it appears to have relatively small contributions to the amplitude of this response.

Using the calculated a-wave model parameters, we subtracted the PIII component from the waveform to isolate the PII component for the no background, 0.01, 0.1, 1 and 10 cd/m^2^ backgrounds. The PII component predominantly results from activity in the ON pathway. This calculation eliminated the large negativity of the waveform in mesopic background conditions. This suggests that the decline in amplitude of the isolated PII component (which was relatively much greater than the decline in PIII amplitude) is likely attributable at least in part to saturation kinetics to maintain retinal sensitivity across increasing background luminance (as discussed above). However, both the waveform narrowing and move to earlier peak times suggest changes in rod signaling pathways with shifts from scotopic to mesopic luminance conditions. The changes in the isolated PII response may indicate rod-driven contributions to the ‘push–pull’ mechanism describing the factors that affect the b-wave (in scotopic and mesopic conditions, mainly driven by ON bipolar cell responses but with influences to the amplitude and shape by OFF bipolar cell responses) [15, 21]. The reduction in activity in the rod BC pathway while there is still a robust rod PIII response may also represent the switching of rod signaling from rod bipolar cells to direct contact with cones or cone bipolar cells in mesopic light levels. This process is thought to be important to prevent saturation of inner retinal pathways by rod responses thus expanding the range of luminances the retina can respond to [18–20].

Although not performed here, drug dissection studies could be used to further assess the contributions from different pathways to the On–Off ERG in the dog, particularly in determining the generators of the drift component. Evidence from drug dissection studies of the On–Off ERG in primates suggests that the positive b-wave is mainly driven by ON bipolar cells whereas the positive d-wave is driven predominantly by the cessation of OFF bipolar cell activity (using L-2-amino-4-phosphonobutyric acid and cis-2,3-piperidine dicarboxylic acid (PDA) to block the activity of the ON and OFF bipolar cells, respectively) [21]. From a comparison of primate and rodent drug dissection studies, it is plausible that the difference in the shape of the Off-response between the species is due to differences in the relative contributions of the ON and OFF pathways. In fact, the On-response appears to be largely similar in both monkey and rat (albeit with some difference in the response shape), whereas the PDA-sensitive component appears to drive the difference between the ERGs of these species, with a very strong corneal-negative component at flash onset and corneal positive component at flash offset in the primate that is not seen in the rodent [27].

## Conclusion

In this study, we designed a protocol with increasing background luminance using long-duration flashes to characterize changes in rod contributions to the On-response of the canine ERG. We showed that the positive PII response saturates at dimmer background luminance than the rod-driven PIII, this may be needed to maintain retinal sensitivity with shifts from scotopic to mesopic lighting. Furthermore, we demonstrated that the rod-driven PIII is responsible for the large negativity present in the On–Off ERG waveforms recorded with mesopic background conditions. This suggests that the shape of the isolated PII indicates potential changes in rod signaling pathways with increasing background luminance. Overall, this study suggests a significant role, and possible changes in signaling, of rod pathways in retinal responses in mesopic background conditions that merit future investigation in dogs and other species.

## Methods

### Animals

Phenotypically normal adults (between 8 months to 2 years of age) that were laboratory beagle crossbreeds from a colony of dogs maintained at Michigan State University were used in this study. The 6 animals (2 males, 4 females) were from a breeding colony used in other unrelated studies. They were housed under 12 h:12 h light:dark cycles. The dogs were tested on a single occasion and subsequently returned to the breeding colony for other unrelated studies. No dogs were excluded from the study.

#### Methods

General anesthesia was induced by intravenous propofol (4–6 mg/kg, PropoFlo, Abbott Laboratories, North Chicago, IL, USA). The animals were intubated and subsequently maintained under anesthesia with isoflurane (IsoFlo, Abbott Laboratories, North Chicago, IL, USA) [between 2–3.5% in a 1-2L/min oxygen flow via a rebreathing circle system for dogs over 10 kg and via a Bain system for dogs under 10 kg].

### Electroretinography (ERG)

General procedures for ERGs were described previously [51]. Pupils were dilated with tropicamide (Tropicamide Ophthalmic Solution UPS 1%, Falcon Pharmaceuticals Ltd., Fort Worth, TX, USA). A monopolar gold-ringed electrode contact lens (ERG-Jet electrode, Fabrinal Eye Care, La Chaux-De-Fonds, CH) was used, and for reference and grounding platinum needle skin electrodes (Grass Technologies, Warwick, RI, USA) were placed 5 mm lateral to the lateral canthus and over the occiput, respectively. ERGs were recorded using an Espion E^2^ Electrophysiology system with ColorDome Ganzfeld (Diagnosys LLC, Lowell, MA).

### ERG protocol

The ERG protocol was designed prior to study onset. A constant flash duration of 250 ms was used with progressively stronger white light background luminance (0, or scotopic, 0.01, 0.1, 1, 10, and 42 cd/m^2^), with 5 different white light stimuli tested at each background (2.5, 25, 180, 500, and 1,250 cd/m^2^) giving a total of 30 steps. Each flash was presented at one second intervals on a dark background and repeated to generate an averaged response detectable against background electrical noise. Dogs were dark adapted for 1 h prior to initiating the protocol, and for 5 min to each subsequent increase in background luminance. A standard short-duration flash (< 4 ms flashes) protocol was also performed on a separate day, with flash stimuli ranging from 0.0002 to 23 cd.s/m^2^ for the dark-adapted ERG and 0.01 to 23 cd.s/m^2^ for the light-adapted (42 cd/m^2^ white background light) ERG – dogs were dark-adapted for 1 h and light-adapted for 10 min, respectively.

### Rod-driven a-wave model

We calculated parameters for the rod-driven a-wave after subtracting photopically matched ERG waveforms [52]. We fit the following equation described by Birch & Hood to the leading edge of the rod a-wave [53, 54]:

$$R(I,t)=(1-exp\lbrack-I\cdot S\cdot{(t-td)}^2\rbrack)\cdot R_{max}\;for\;t>t_d$$

The amplitude *R* is a function of the retinal luminance *I* and time *t* after the flash onset and *t*_*d*_ is a brief delay. *S* is a sensitivity factor and *R*_*max*_ is the maximum amplitude of the response.

A-wave model parameters were analyzed using a repeated-measures ANOVA with a post-hoc Tukey HSD performed to determine significant between-group comparisons.

### Curve fitting

We calculated parameters using the *lmfit* curve-fitting program in the Python 3.6 environment [55], using the Levenberg–Marquardt algorithm to calculate optimal parameter values via least squares minimization [56]:

$$f({{\varvec{X}}}_{i},{\varvec{\beta}}+{\varvec{\delta}}) \approx f({{\varvec{X}}}_{i},{\varvec{\beta}}) + {{\varvec{J}}}_{{\varvec{i}}}\boldsymbol{ }{\varvec{\delta}}$$

where **J**_***i***_ is the gradient of *f* with respect to ***β***. Successive calculation of the parameter ***δ ***that minimizes the sum of square of the residuals *S* is performed computationally until final model parameters are obtained [57, 58].

We determined model goodness-of-fit with the least-squares parameter, with values less than 0.25 considered a good fit [54]:

$$lsq= \frac{\sum_{i=1}^{n}{({y}_{i}-f\left({X}_{I},{\varvec{\beta}}\right))}^{2}}{\sum_{i=1}^{n}{({y}_{i}-mean\left(y\right))}^{2}}$$

### Isolating rod-driven PII

The parameters calculated from the a-wave model were used to define the rod-driven PIII. This modeled response was then subtracted from the photopically subtracted waveforms described above to isolate the postreceptoral PII process for responses with a measurable a-wave (0, 0.01, 0.1, and 1 cd/m^2^ background light levels).

## Data Availability

All data generated or analyzed during this study are included in this published article. The datasets for each individual dog during the current study are available from the corresponding author on reasonable request.

## References

[1] Burns ME, Baylor DA (2001). Activation, deactivation, and adaptation in vertebrate photoreceptor cells. Annu Rev Neurosci.

[2] Arshavsky VY, Lamb TD, Pugh EN (2002). G Proteins and Phototransduction. Annu Rev Physiol.

[3] Hagins WA, Penn RD, Yoshikami S (1970). Dark Current and Photocurrent in Retinal Rods. Biophys J.

[4] Fain GL, Matthews HR, Cornwall MC, Koutalos Y (2001). Adaptation in vertebrate photoreceptors. Physiol Rev.

[5] Hood DC, Birch DG (1995). Phototransduction in human cones measured using the a-wave of the ERG. Vision Res.

[6] Kraft TW, Schneeweis DM, Schnapf JL (1993). Visual transduction in human rod photoreceptors. J Physiol.

[7] Thomas MM, Lamb TD (1999). Light adaptation and dark adaptation of human rod photoreceptors measured from the a-wave of the electroretinogram. J Physiol.

[8] Euler T, Haverkamp S, Schubert T, Baden T (2014). Retinal bipolar cells: elementary building blocks of vision. Nat Rev Neurosci.

[9] Nelson R, Kolb H. ON and OFF pathways in the vertebrate retina and visual system. The visual neurosciences 2004;1:260-278.

[10] Wässle H (2004). Parallel processing in the mammalian retina. Nat Rev Neurosci.

[11] Duvoisin RM, Morgans C, Taylor W (2005). The mGluR6 receptors in the retina: Analysis of a unique G-protein signaling pathway. Cellscience Rev.

[12] Thoreson WB (2007). Kinetics of synaptic transmission at ribbon synapses of rods and cones. Mol Neurobiol.

[13] Rufiange M, Rousseau S, Dembinska O, Lachapelle P (2002). Cone-dominated ERG luminance–response function: the Photopic Hill revisited. Doc Ophthalmol.

[14] Wali N, Leguire LE (1992). The photopic hill: A new phenomenon of the light adapted electroretinogram. Doc Ophthalmol.

[15] Sieving PA, Murayama K, Naarendorp F (1994). Push-pull model of the primate photopic electroretinogram: a role for hyperpolarizing neurons in shaping the b-wave. Vis Neurosci.

[16] Bloomfield SA, Dacheux RF (2001). Rod vision: pathways and processing in the mammalian retina. Prog Retin Eye Res.

[17] Deans MR, Volgyi B, Goodenough DA, Bloomfield SA, Paul DL (2002). Connexin36 is essential for transmission of rod-mediated visual signals in the mammalian retina. Neuron.

[18] Hornstein EP, Verweij J, Li PH, Schnapf JL (2005). Gap-junctional coupling and absolute sensitivity of photoreceptors in macaque retina. J Neurosci Off J Soc Neurosci.

[19] Schneeweis DM, Schnapf JL (1995). Photovoltage of rods and cones in the macaque retina. Science.

[20] Verweij J, Dacey DM, Peterson BB, Buck SL (1999). Sensitivity and dynamics of rod signals in H1 horizontal cells of the macaque monkey retina. Vision Res.

[21] Bush RA, Sieving PA (1994). A proximal retinal component in the primate photopic ERG a-wave. Invest Ophthalmol Vis Sci.

[22] Robson JG, Saszik SM, Ahmed J, Frishman LJ (2003). Rod and cone contributions to the a-wave of the electroretinogram of the macaque. J Physiol.

[23] Cameron AM, Mahroo OAR, Lamb TD (2006). Dark adaptation of human rod bipolar cells measured from the b-wave of the scotopic electroretinogram. J Physiol.

[24] Robson JG, Maeda H, Saszik SM, Frishman LJ (2004). In vivo studies of signaling in rod pathways of the mouse using the electroretinogram. Vision Res.

[25] Naarendorp F, Williams GE (1999). The *d* -wave of the rod electroretinogram of rat originates in the cone pathway. Vis Neurosci.

[26] Xu X, Karwoski C (1995). Current source density analysis of the electroretinographic d wave of frog retina. J Neurophysiol.

[27] Lei B (2003). The ERG of guinea pig (Cavis porcellus): comparison with I-type monkey and E-type rat. Doc Ophthalmol.

[28] Kondo M, Miyake Y, Horiguchi M, Suzuki S, Tanikawa A (1998). Recording Multifocal Electroretinogram On and Off Responses in Humans. Invest Ophthalmol Vis Sci.

[29] Ueno S, Kondo M, Ueno M, Miyata K, Terasaki H, Miyake Y (2006). Contribution of retinal neurons to d-wave of primate photopic electroretinograms. Vision Res.

[30] Granit R (1933). The components of the retinal action potential in mammals and their relation to the discharge in the optic nerve. J Physiol.

[31] Müller F, Kaupp UB (1998). Signal transduction in photoreceptor cells. Naturwissenschaften.

[32] Green DG (1973). Scotopic and photopic components of the rat electroetinogram. J Physiol.

[33] Granit R, Therman PO (1935). Excitation and inhibition in the retina and in the optic nerve. J Physiol.

[34] Wündsch L, Lützow AV (1971). The effect of aspartate on the ERG of the isolated rabbit retina. Vision Res.

[35] Vinberg FJ, Strandman S, Koskelainen A (2009). Origin of the fast negative ERG component from isolated aspartate-treated mouse retina. J Vis.

[36] Arden GB (1976). Voltage gradients across the receptor layer of the isolated rat retina. J Physiol.

[37] Hanawa I, Tateishi T (1970). The effect of aspartate on the electroretinogram of the vertebrate retina. Experientia.

[38] Veske A, Nilsson SE, Narfström K, Gal A (1999). Retinal dystrophy of Swedish briard/briard-beagle dogs is due to a 4-bp deletion in RPE65. Genomics.

[39] Kondo M, Das G, Imai R, Santana E, Nakashita T, Imawaka M (2015). A Naturally Occurring Canine Model of Autosomal Recessive Congenital Stationary Night Blindness. PLoS ONE.

[40] Somma AT, Moreno JCD, Sato MT, Rodrigues BD, Bacellar-Galdino M, Occelli LM (2017). Characterization of a novel form of progressive retinal atrophy in Whippet dogs: a clinical, electroretinographic, and breeding study. Vet Ophthalmol.

[41] Marinho LLP, Occelli LM, Pasmanter N, Somma AT, Montiani-Ferreira F, Petersen-Jones SM (2019). Autosomal recessive night blindness with progressive photoreceptor degeneration in a dog model. Invest Ophthalmol Vis Sci.

[42] Petersen-Jones SM, Occelli LM, Winkler PA, Lee W, Sparrow JR, Tsukikawa M (2018). Patients and animal models of CNGβ1-deficient retinitis pigmentosa support gene augmentation approach. J Clin Invest.

[43] Occelli LM, Schön C, Seeliger MW, Biel M, Michalakis S, Petersen-Jones S, et al. Gene Supplementation Rescues Rod Function and Preserves Photoreceptor and Retinal Morphology in Dogs, Leading the Way Towards Treating Human PDE6A-Retinitis Pigmentosa. Hum Gene Ther. 2018;28(12)1189-201. 10.1089/hum.2017.155 29212382

[44] Petersen-Jones SM, Komáromy AM (2015). Dog models for blinding inherited retinal dystrophies. Hum Gene Ther Clin Dev.

[45] Pasmanter N, Petersen-Jones SM (2020). A review of electroretinography waveforms and models and their application in the dog. Vet Ophthalmol.

[46] Oh A, Loew ER, Foster ML, Davidson MG, English RV, Gervais KJ, Herring IP, Mowat FM (2018). Phenotypic characterization of complete CSNB in the inbred research beagle: how common is CSNB in research and companion dogs?. Doc Ophthalmol.

[47] Donner K (1992). Noise and the absolute thresholds of cone and rod vision. Vision Res.

[48] Dunn FA, Doan T, Sampath AP, Rieke F (2006). Controlling the gain of rod-mediated signals in the Mammalian retina. J Neurosci Off J Soc Neurosci.

[49] Frishman LJ, Robson JG, Reddy MG (1996). Effects of background light on the human dark-adapted electroretinogram and psychophysical threshold. J Opt Soc Am A.

[50] Shapley R, Enroth-Cugell C (1984). Chapter 9 Visual adaptation and retinal gain controls. Prog Retin Res.

[51] Annear MJ, Bartoe JT, Barker SE, Smith AJ, Curran PG, Bainbridge JW (2011). Gene therapy in the second eye of RPE65-deficient dogs improves retinal function. Gene Ther.

[52] Brigell M, Jeffrey BG, Mahroo OA, Tzekov R (2020). ISCEV extended protocol for derivation and analysis of the strong flash rod-isolated ERG a-wave. Doc Ophthalmol.

[53] Hood DC, Birch DG (1996). Assessing abnormal rod photoreceptor activity with the a-wave of the electroretinogram: Applications and methods. Doc Ophthalmol.

[54] Hood DC, Birch DG (1990). The A-wave of the human electroretinogram and rod receptor function. Invest Ophthalmol Vis Sci.

[55] Van Rossum G, Drake FL. Python 3 Reference Manual. Scotts Valley: CreateSpace; 2009.

[56] Newville M, Stensitzki T, Allen DB, Ingargiola A. LMFIT: Non-Linear Least-Square Minimization and Curve-Fitting for Python. Zenodo; 2014 [cited 2020 Jan 20]. Available from: https://zenodo.org/record/11813

[57] Levenberg K (1944). A method for the solution of certain non-linear problems in least squares. Q Appl Math.

[58] Marquardt DW (1963). An Algorithm for Least-Squares Estimation of Nonlinear Parameters. J Soc Ind Appl Math.

